# Pineal gland senescence: an emerging ageing-related pathology?

**DOI:** 10.1007/s42000-025-00720-9

**Published:** 2025-12-16

**Authors:** Emma Short, Ramzi Ajjan, Thomas M Barber, Sunil Bhandari, Paul Chazot, Jennifer L. Garrison, Anushka Goyal, Robert Huckstepp, Noordin Jamal, Venkateswarlu Kanamarlapudi, Alpar Lazar, Thomas Lee, Adriana A. S. Tavares, Jeremy J Tree, Jack Wellington, Stuart RG Calimport, Barry Bentley

**Affiliations:** 1 Cardiff School of Technologies, Cardiff Metropolitan University, Cardiff, UK; 2 Department of Cellular Pathology, Swansea Bay University Health Board, Swansea, UK; 3 Leeds Institute of Cardiovascular and Metabolic Medicine, University of Leeds, Leeds, UK; 4 Warwickshire Institute for the Study of Diabetes, Endocrinology and Metabolism, University Hospitals Coventry and Warwickshire, Clifford Bridge Road, Coventry, UK; 5 Division of Biomedical Sciences, Warwick Medical School, University of Warwick, Coventry, UK; 6 Department of Renal Medicine, Hull University Teaching Hospitals NHS Trust, Hull, UK; 7 Hull York Medical School, East Yorkshire, UK; 8 Durham University, Durham, UK; 9 Center for Healthy Aging in Women, Buck Institute for Research on Aging, CA Novato, USA; 10 Bristol Medical School, Bristol, UK; 11 School of Life Sciences, University of Warwick, Coventry, UK; 12 University of East Anglia, Norwich, UK; 13 Swansea University Medical School, Swansea University, Swansea, UK; 14 Centre for Cardiovascular Science, University of Edinburgh, Edinburgh, UK; 15 Edinburgh Imaging, University of Edinburgh, Edinburgh, UK; 16 Director of Advanced Diagnostics and Medical Technologies Research Institute, Swansea University, Swansea, UK; 17 Leeds Teaching Hospital NHS Foundation Trust, Leeds, UK; 18 Collaboration for the Advancement of Sustainable Medical Innovation (CASMI), University College London, London, UK; 19 Center for Engineering in Medicine and Surgery, Harvard Medical School, Boston, MA USA; 20 Department of Surgery, Massachusetts General Hospital, Harvard Medical School, Boston, MA USA; 21 Shriners Children’s, Boston, MA USA

**Keywords:** Pineal gland, Ageing, Senescence, Ageing-related pathology, Pineal gland senescence

## Abstract

An ageing-related pathology has recently been described as one that develops and/or progresses with increasing chronological age, that is associated with, or contributes to, functional decline and that is evidenced by studies in humans. The pineal gland is a photo-neuroendocrine organ whose primary function is to produce and secrete melatonin in response to light-dark cycle environmental cues. The gland may undergo ageing-related structural and morphological changes, including calcification, gliosis, cyst formation, and reduced density of β-adrenergic receptors, which are hypothesised to reduce melatonin secretion. Pineal gland senescence describes the ageing-related decline in neuroendocrine function, with reduced secretion of melatonin, which may contribute to ageing-related sleep disorders and disruption of other circadian-driven physiological functions and may have secondary effects such as contributing to cognitive and mood disorders related to sleep disturbance.

## Introduction

Around the world people are living longer with virtually all countries experiencing a growth in both the number and proportion of older people in the population [1]. Ageing is characterised inter alia by the time-related progressive accumulation of damage and dysfunction, which can occur at molecular, cellular, tissue, organ, and organ system levels [2]. This can have detrimental effects on an individual’s intrinsic capacity and can negatively impact physiological, cognitive, psychological and social functioning, and/or socioeconomic status and productivity levels [2]. Although there have been significant advances in our understanding of the biological features of ageing, these insights have not yet been translated into clinically relevant classification and staging systems for ageing-related pathologies, diseases, or syndromes [2].

In 2019, Calimport et al. called for the systematic and comprehensive classification and staging of such ageing-related pathologies at the metabolic, tissue, organ, and systemic levels [3]. To address this, the International Consortium for the Classification of Ageing-Related Pathologies (ICCARP) was established in 2023, led by Cardiff Metropolitan University [2]. The ICCARP has recently defined the criteria for an ageing-related pathology as one that [2]:

- Develops and/or progresses with increasing chronological age.
- Should be associated with, or contribute to, functional decline or an increased susceptibility to functional decline.
- Is evidenced by studies in humans.

Here we review the evidence to determine whether pineal gland senescence should be recognised as an ageing-related pathology. It is vital to develop comprehensive classification systems for ageing-related pathologies as this will allow societies to better identify, quantify, understand, and meet the healthcare, workforce, wellbeing, and socioeconomic needs of ageing populations while supporting the development and utilisation of interventions to prevent or to slow, halt, or reverse the progression of ageing-related pathologies [2].

## Anatomy, histology, and function of the pineal gland

The pineal gland is a photo-neuroendocrine gland located in the midline of the brain outside the blood-brain barrier [4, 5]. It is part of the epithalamus, is attached to the third ventricle by a Short stalk, and can weigh up to 180 g [4, 5]. Its primary role is to receive information about the light-dark cycle from the environment, which it responds to through the production and secretion of melatonin [4, 5]. Photosensory information reaches the pineal gland through a multineuronal pathway that originates in the retina [4]; melatonin is secreted by pinealocytes, the predominant cell type of the gland [4]. The pineal gland is derived from vimentin expressing Pax6-positive, radially aligned neuroepithelial cells of the third ventricle, which eventually form the pinealocytes and astroglia [6]. In addition to glial tissue, in particular astrocytes [4, 5], the tissue is also colonised by microglia [6] and contains networks of neurons and blood vessels necessary for its function [7]. The innervation of the pineal gland is primarily sympathetic, originating from the superior cervical ganglia [7]. It receives its major arterial supply from the pineal arteries which are largely derived from the medial posterior choroidal arteries [5, 8], with venous drainage occurring via the lateral pineal veins and the cerebral vein of Galen [8].

Photic information is relayed from the retina to the suprachiasmatic nucleus (SCN) of the hypothalamus and then to other hypothalamic regions [5]. Retinal ganglion cells that contain melanopsin and have an intrinsic photoreceptor capability play an important role in this process [5].

When it is light, the SCN secretes gamma-amino butyric acid (GABA), which in turn inhibits neurons in the paraventricular nucleus (PVN) of the hypothalamus. In darkness, the SCN secretes glutamate, which activates pathways from parvocellular pre-autonomic neurons of the PVN via the superior cervical ganglion to stimulate melatonin production by the pineal gland in response to noradrenaline [5]. In continuous darkness or dim light, the SCN acts as an endogenous oscillator [5] with a fixed near 24-hour period generated by a cell-autonomous molecular clock [9]. Other less significant factors that may modulate melatonin production and secretion include posture, exercise, certain medications, alcohol, and caffeine [5].

**Fig. 1:**
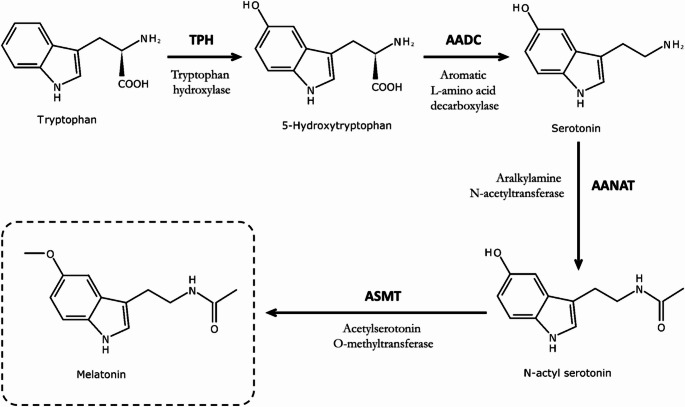
The conversion of tryptophan to serotonin

Melatonin, a significant portion bound to albumin, is released from the pineal gland directly into cerebrospinal fluid (CSF) and into the peripheral circulation [5]. It has a half-life of approximately 40 min and is metabolised in the liver prior to urinary excretion [5]. The precursor to melatonin is L-tryptophan, which is taken up from the circulation and converted to 5-hydroxytryptophan by tryptophan hydroxylase (TPH) before being converted to serotonin (5-HT) by the aromatic L-amino acid decarboxylase enzyme (AADC) [10]. 5-HT is metabolised by the rate-limiting enzyme arylalkylamine N-acetyltransferase (AANAT) to N-acetyl-5-hydroxytryptamine and in turn by hydroxyindole-O-methyltransferase (HIOMT) (also known as N-acetylserotonin methyltransferase, or ASMT) to melatonin [10] (Fig. 1).

Melatonin is a signal of darkness and, although one of its major functions is to promote sleep, it has several other vital roles. Melatonin secretion is a robust biochemical signal of night and plays a role in the maintenance of circadian rhythms, for example, those of core body temperature, the immune system, and regulation of glucose metabolism. Melatonin has additional functions, including antioxidant effects, neuroprotection, sex hormone regulation, modulating thyroid and thymic function, thermoregulation and cardiovascular physiology, exerting antitumour effects, acting as an antisenescence molecule, and even in modulating thrombosis risk [4, 5, 10–12].

## Pineal gland senescence

We, the ICCARP Endocrine and Metabolic Working Group, hypothesise that pineal gland senescence may represent an ageing-related pathology as it describes an ageing-related decline in pineal gland functioning. This causes a reduction in the secretion of melatonin that may contribute to ageing-related sleep disorders as well as other physiological, cognitive, and psychiatric dysfunctions related to disturbances in circadian rhythm and melatonin concentrations. The current paper will describe the pathophysiology of the pineal gland and will discuss whether pineal gland senescence should be considered as a diagnostic entity.

Melatonin concentrations appear to be relatively stable from the late teens until age 35–40 years, or possibly even earlier, after which there is an ageing-related reduction in the amplitude of melatonin secretion such that the maximal nocturnal peak concentration of melatonin declines [10, 13, 14]. Reduced melatonin concentrations during ageing, especially nocturnal levels, have been reported in the pineal gland, plasma, cerebrospinal fluid (CSF), urine (as 6-hydroxy melatonin), and saliva [10, 15, 16]. Therefore, in many elderly individuals, the day/night differences in melatonin secretion are almost absent [17]. It has been reported that by the age of 60 years, the day/night difference in melatonin is 80% lower than that seen in teenage years, the difference being negligible in later decades [18–21]. This may contribute to the increased prevalence of sleep-related disorders that occur with ageing [22]. Melatonin secretion during ageing inversely correlates with sleep disturbances [17], and it is recognised that deviations from the normal pattern of melatonin secretion are associated with sleep disturbances [13]. Much of the literature on the types of sleep disturbances associated with melatonin disruption, such as sleep latency, sleep-wake disturbance, and poor sleep quality, are contained within the International Classification of Sleep Disturbances − 3 (ICSD-3) categories of circadian rhythm sleep-wake disorder (CRSWD) and insomnia [23, 24].

In addition to sleep disorders [25, 26], the disruption of melatonin secretion with associated sleep disturbance has been observed across a range of clinical conditions, including diabetes, neurodevelopmental disorders, and neurological diseases [19, 27–31]. For example, circadian rhythm dysregulation is a prominent feature of Alzheimer’s disease, and melatonin levels are often less than that of healthy control subjects [10, 19, 27]. Similarly, sleep problems are often a non-motor symptom in Parkinson’s disease, studies having shown that in this disorder, there is a correlation between decreased rapid eye movement (REM)/slow wave sleep and a reduction in melatonin output, as well as substantially impaired secretion amplitudes in comparison to healthy controls. Patients with Parkinson’s disease who experience excessive daytime sleepiness were also found to have more prominent dysfunction of circadian melatonin output than patients without somnolence [30, 31].

Whilst ageing and the associated disruption of the circadian rhythm have been identified as a significant risk factor for insomnia [32], it is important to highlight that there are many other factors that play a role, for example, comorbid medical conditions, including psychiatric disorders, pain [32], incontinence, and various medications.

## Pathophysiology of pineal gland senescence

**Fig. 2:**
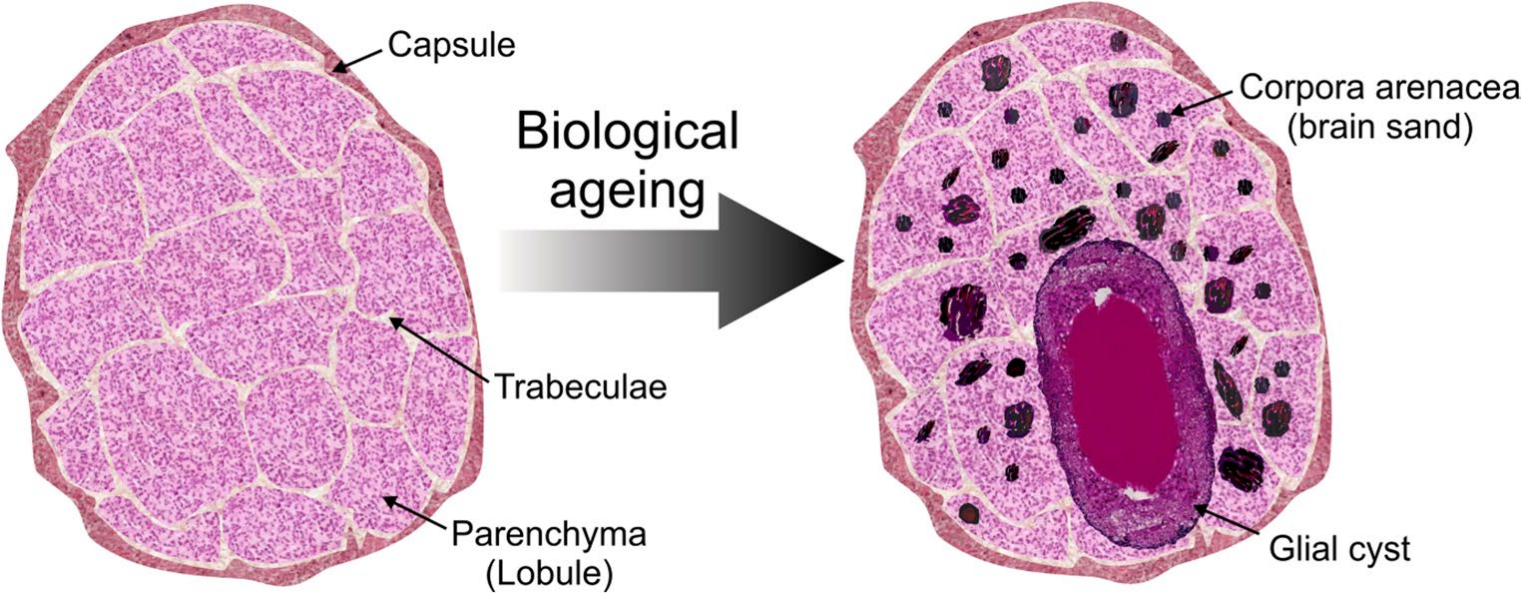
Morphological changes in the pineal gland with increasing age. The pineal gland, which is encapsulated in a pial capsule, is largely composed of pinealocytes and glia. The gland is separated into lobules by trabeculae. With increasing age there is increasing calcification (corpora arenacea, ‘brain sand’), and there may also be a reduction in the volume of the gland, gliosis, glial cyst formation and reduced density of β-adrenergic receptors.

Pineal gland senescence results from several structural and morphological changes (Fig. 2). Primarily, the structures of the pineal gland calcify with increasing age, forming concretions known as corpora arenacea [33]. The presence and degree of gland calcification can be determined through neuroimaging techniques, such as computed tomography (CT) scanning [34], positron emission tomography (PET) scanning [35], or magnetic resonance imaging (MRI) [36]. Corpora arenacea are composed largely of calcium and phosphorus, with smaller amounts of magnesium and strontium [37]. Calcification can occur within the capsule (extra-pineal calcification) and/or the parenchyma of the gland (pineal calcification) [37]. The prevalence of calcification seems to increase with advancing age. One study reported that calcification began around 30 years of age [38] and then stabilised between the fifth and seventh decades [38, 39], describing an absence of pineal gland calcification in the 0–25 age group, increasing to 14% in the 46–65 age group, and 15% in the 66–96 age group [38]. However, pineal gland calcification has also been identified in children, with a prevalence of 1% in those younger than 6 years, 8% in those under the age of 10 years, and in 39% of those aged between 8 and14 years [40]. Although pineal gland calcification may be observed in younger individuals, a recent systematic review [37] stated that increasing chronological age is associated with an increased prevalence of pineal gland calcification.

Characteristics or risk factors that can be associated with an increased prevalence of pineal gland calcification include white ethnicity and smoking, while sex differences require further investigation [37–39].

The mechanisms of pineal gland calcification have not been fully elucidated, but several hypotheses have been proposed, namely, the following:

1. Mast cell infiltration and release of tryptase [41], which can cause the release and build-up of intracellular calcium in cells expressing receptors such as protease-activated receptor 2 (PAR2) [42].
2. Pineal polypeptides are extruded into the extracellular space with a carrier protein, neuroepiphysin, in exchange for calcium, resulting in calcium deposits that are related to the secretory function of the gland [41].
3. The cytoplasmic matrix, vacuoles, mitochondria, and endoplasmic reticulum of pinealocytes are intracellular calcification sites that become calcified through the addition of hydroxyapatite crystals in the context of a hypercalcaemic intracellular milieu due to a failure of Ca^2+^-ATPase pumps [41].
4. Pineal calcification may result from the activity of osteoblast-like cells which differentiate from mesenchymal stem cells (MSCs) [41].

Some studies have reported that plasma melatonin levels and/or urinary metabolites show no relationship to pineal calcification [43, 44]. However, it has been demonstrated that the amount of uncalcified pineal tissue is positively associated with urinary melatonin metabolites [45]. Furthermore, the degree of pineal calcification is negatively associated with sleep-related parameters such as REM sleep percentage, total sleep time, and sleep efficiency [45], with uncalcified pineal volume correlating negatively with sleep rhythm disturbances [46]. Pineal gland calcification is significantly associated with the presence of daytime tiredness and sleep disturbance, thus supporting the hypothesis that pineal gland calcification impairs melatonin production, which can lead to disturbed circadian rhythm in the sleep-wake cycle [47]. Of note, pineal gland calcification may be associated with neurodegenerative and psychiatric disorders [37, 38, 41] in addition to its putative role in sleep disturbances, and melatonin is thought to play a key role in this. We therefore suggest that the neuroendocrine changes that are associated with sleep disorders with increasing age can have secondary consequences, potentially accelerating an ageing-related decline in cognitive function and/or amplifying mood disorders.

It is plausible that it is not the absolute volume of calcium deposits that impair melatonin secretion per se, but rather the proportion of the pinealocyte parenchyma replaced by such deposits. Indeed, pineal parenchyma volume positively correlates with urinary 6-sulfatoxymelatonin levels [44], while uncalcified pineal volume correlates with salivary and plasma melatonin levels [46, 48].

Although there is to date no convincing evidence that pineal gland volume decreases linearly with advancing age [38, 44], ageing-related changes have been reported, with the gland reaching peak volume between the ages of 46–65 years, after which size tended to decrease in the cohorts studied. This U-shaped curve in the relationship between pineal gland volume and age does not necessarily correspond to a similar functional profile as it is feasible that the gland’s secretory capacity is compromised by active pinealocytes affected by ageing-related calcification. There are reports of pinealocyte number reduction with chronological ageing, but this remains controversial in both humans [49, 50] and other species [51–53]. However, studies on pineal gland volume have largely used manual delineation, have generally included a limited number of individuals, and have not always explored a wide age distribution. More recent work employing MRI structural atlas data from 295 individuals aged between 18 and 75 years has demonstrated a negative correlation between chronological age and pineal gland volume, indicating a general shrinkage of the gland with ageing. However, volume reduction also occurs in other parts the brain, indicating that this is a global process and not related only to the pineal gland per se [54]. However, pineal gland volume does not necessarily reflect function, which can be affected by structural changes associated with ageing.

Astrocytes play an important role in supporting pinealocytes and in providing structural support for the gland [55]. There are two types of glial cells present. The first are vimentin positive fibrous astrocytes (type 1 pineal glia) that are found in the trabeculae surrounding the lobules. They have perivascular feet associated with blood vessels [55, 56]. The second type of glial cells are glial fibrillary acidic protein (GFAP) positive protoplasmic astrocytes (type 2 pineal glia) that occur throughout the lobules, and they have processes/fibrils/plexuses wrapped around blood vessels [55, 56]. Both vimentin- and GFAP-positive fibres wrap around corpora arenacea to insulate neurons from them, although fibrous astrocytes only do so within trabeculae [56].

Whilst some papers describe an increased proportion of glia within the pineal gland with increasing age [57, 58], this is not a consistent finding [38]. Furthermore, although it has previously been proposed that the prevalence of glial cysts increases with age [59], this is not always supported by the literature [38]. Glial cysts are commonly associated with headaches, visual issues, and impaired eye movement, particularly loss of upward gaze [60]. They have been reported in association with dementia and are more prevalent in patients with pancreatic dysfunction, adrenal gland adenomas, and thyroid goitre and less prevalent in hypertensive patients [61]. The prevalence and quantification of gliosis and cyst formation need to be better characterised.

**Fig. 3:**
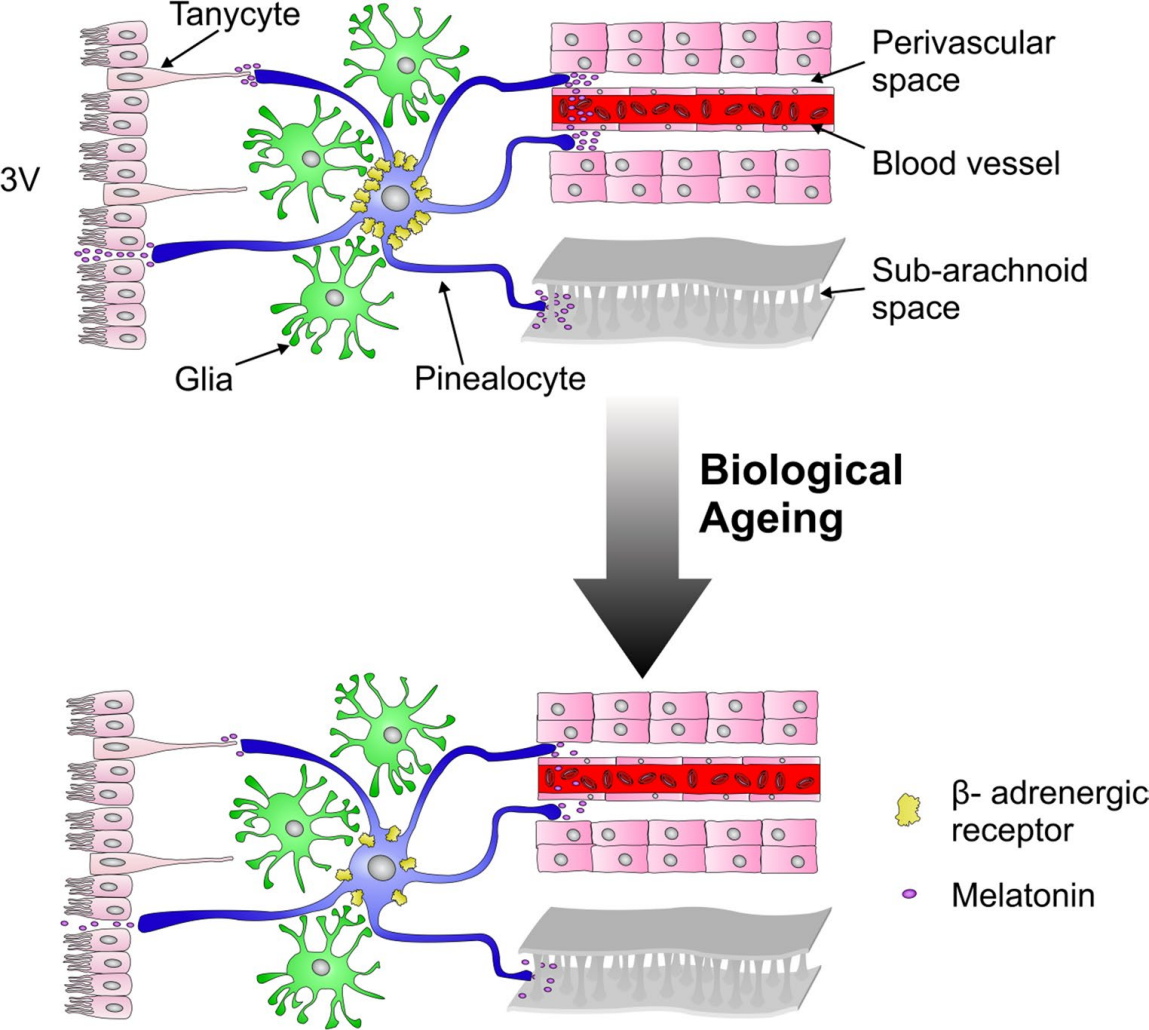
Autonomic dysregulation is associated with reduced melatonin secretion with increasing age. Stimulation of pinealocytes (blue) by adrenergic receptors (yellow) induces the release of melatonin (purple) into the perivascular space or subarachnoid space. With increasing age, there is a reduction in adrenergic receptor density and altered receptor function, leading to decreased melatonin release (3 V: 3rd ventricle).

Additional features of the ageing pineal gland that may have a negative impact on melatonin synthetic capacity include reduced density of β-adrenergic receptors (Fig. 3) and downregulation of the expression of genes whose protein products participate in the synthesis of melatonin [41].

Melatonin release from the pineal gland requires sympathetic input from the superior cervical ganglion and is under the control of postsynaptic β-adrenoceptors which induce cAMP dependent activation of PKA and ultimately the synthesis of enzymes in the melatonin pathway [62]. This pathway is greatly enhanced by postsynaptic α-adrenoceptors, which potentiate calcium influx and drive protein synthesis through cAMP, cGMP, and PKC pathways [63]. Animal studies have reported that stimulation of melatonin release in response to β-adrenoceptor activation wanes with increasing age [64]. This can is often associated with an ageing-dependent reduction in receptor density [65, 66] and a change in receptor function [67]. Interestingly, β-adrenoceptor density appears to be inversely proportional to the sympathetic drive to the pineal gland [68]; therefore, a loss of receptor density may be associated with an ageing-related increase in sympathetic drive.

In addition to primary pineal gland senescence, an ageing-related reduction in melatonin secretion can also be due to secondary factors. Though these are outside the scope of this paper, such factors include reduced light exposure with increasing age, visual defects which impair the capacity to detect light, lesions or atrophy of the SCN, PVN, or superior cervical ganglia, autonomic dysregulation with subsequent impairment in noradrenergic gland stimulation, and certain medications [5, 13, 41, 69].

There is limited research into the environmental factors that contribute to biological ageing of the pineal gland. However, associations have been reported between pineal gland calcification and sunlight exposure, low altitude, and fluoride exposure [70–73]. It is of importance to investigate and determine the relationships between environmental factors, genomic factors, lifestyle factors, and pineal gland ageing and how they interact with currently recognised ageing-related diseases, disorders, and syndromes.

## Is pineal gland senescence an ageing-related pathology?

Pineal gland senescence is an emerging entity which appears to fulfil the criteria for an ageing-related pathology, primarily for the following reasons:

- Morphological changes are observed with increasing chronological age, in particular pineal gland calcification.
- Such changes seem to be associated with impaired melatonin secretion.
- These findings have been reported in studies carried out in humans.

However, further evidence is required before pineal gland senescence can be definitively regarded as such. Many studies have reported conflicting or non-significant results, for example, in the nature of the relationship between pineal gland calcification and melatonin secretion, as to whether gliosis occurs with increasing biological age, and precisely how pinealocyte number correlates with biological age of the pineal gland.

## Summary

Our hypothesis is that structural, morphological, and functional changes of the pineal gland develop and progress with increasing age and that these changes cause a progressive reduction in the secretion of melatonin. It is important to obtain further evidence in order to better characterise the degenerative changes of the pineal gland, their epidemiology, and how they are related to ageing. It is also vital to determine which changes impact melatonin production and/or secretion and how they do so as well as to elucidate the aetiology of pineal gland calcification.

If pineal gland senescence is to be considered as an ageing-related pathology, methods for its diagnosis and staging would need to be determined. This could then lead to opportunities to develop interventions to halt, reverse, or slow its progression, to improve the quality of life of affected individuals, and to promote healthy longevity.
